# A resource-oriented intervention addressing balance in everyday activities and quality of life in people with advanced cancer: protocol for a feasibility study

**DOI:** 10.1186/s40814-022-01038-8

**Published:** 2022-04-20

**Authors:** Marc Sampedro Pilegaard, Helle Timm, Heidi Klit Birkemose, Sandra Bakkegaard Dupont, Dorthe Soested Joergensen, Karen la Cour

**Affiliations:** 1 REHPA, the Danish Knowledge Centre for Rehabilitation and Palliative Care, Odense University Hospital, 5700 Nyborg, Denmark; 2 The Research Unit for User Perspectives and Community-based Interventions, the Research Group for Occupational Science, Department of Public Health, University of Southern Denmark, 5000 Odense C, Denmark; 3 The National Institute of Public Health, University of Southern Denmark, Copenhagen K, Denmark; 4 Copenhagen Centre for Cancer and Health, Municipality of Copenhagen, 2200 Copenhagen N, Denmark

**Keywords:** Neoplasms, Everyday activities, Quality of life, Rehabilitation, Palliative care, Feasibility study, Intervention development

## Abstract

**Background:**

People with advanced cancer need to balance their resources and energy in order to experience enjoyment and quality of life in the time they have left. A resource-oriented intervention is developed targeting these aspects. The present protocol presents a feasibility study of this resource-oriented intervention in people with advanced cancer.

**Methods:**

A feasibility study with a repeated-measurement design without a control group will be conducted at the research clinic of REHPA, the Danish Knowledge Center for Rehabilitation and Palliative Care. Data will be gathered at baseline, during and after a 5-day residential stay, after 6 weeks, during a 2-day follow-up stay and after 12 weeks. In total, 20–25 home-living adults (≥ 18 years) with advanced cancer reporting needs in everyday life will be recruited. The intervention consists of workshops and engagement in physical and creative everyday activities provided by a multidisciplinary team.

Outcome measures are quality of life, physical function and fatigue, which will be assessed using the European Organisation for Research and Treatment of Cancer Quality-of-Life Questionnaire Core-30. Balance in everyday activities will be assessed using the Occupational Balance Questionnaire.

Feasibility data will also be collected regarding (1) fidelity, (2) adherence, (3) dose and (4) reach and mechanisms of impact. For exploration of mechanism of impact, participant observations and focus group interviews will be used.

**Discussion:**

This study presents a new approach in rehabilitation and palliative care aimed at supporting people with advanced cancer; instead of identification of problems, the present resource-oriented palliative rehabilitation intervention will target people’s resources, enhancing balance in everyday activities and underpinning enjoyment and quality of life. The results from the feasibility study can inform ways in which to support the everyday life of people with advanced cancer and thus have potential to improve their quality of life. The long-term perspectives are to evaluate the intervention in terms of effect, process and cost-effectiveness. This will provide evidence to adjust the content of rehabilitation and palliative care for this group of people.

**Trial registration:**

NCT04772690

*Name of the registry*: Balance, Activity and Quality of Life (BAL)

*Date of registry*: February 26, 2021

## Key messages regarding feasibility

- The content, delivery and outcomes of a newly developed resource-oriented intervention for people with advanced cancer should be feasibility tested.
- The present study presents a protocol for a feasibility study evaluating a resource-oriented intervention.
- The present feasibility study may contribute with important knowledge to further develop the contents and delivery of a resource-oriented palliative rehabilitation intervention.

## Background

People with advanced cancer wish to manage everyday activities and experience enjoyment in their everyday lives [1, 2]. Furthermore, they want to prioritise spending time with their families (social activities and relations), to remain mobile and to participate in community and recreative activities to the extent possible [3–5]. Committing to and participating in these activities are of great importance, enhancing feelings of autonomy, dignity and well-being [6–8].

Research shows that people with advanced cancer often suffer from fatigue and find it difficult to prioritise their energy and strength in a manner allowing them to manage activities that are important to them and to achieve a balanced mix of everyday activities [3, 4, 8]. Balancing everyday activities is defined as the subjective experience of having achieved the right mix (amount and variation) of activities in one’s activity pattern [9]. A cross-sectional study by Wæhrens et al. showed that people with advanced cancer spent most of their time on managing activities of daily living, leaving less energy and strength for other joyful everyday activities [4]. Social and physical activities may contribute to enjoyment, i.e. by engaging in craft, listening to music and being and walking in the nature. These activities are important because they can divert attention from illness and problems and contribute to enjoyment and quality of life [10, 11]. A process evaluation from a full-scale randomized controlled trial (RCT) found that people with advanced cancer had a preference for interventions that focus on their resources and contribute to enjoyment than interventions focusing on problems and activities they can no longer conduct [12]. This is in line with findings from a pilot study, which showed that people with advanced cancer wanted more enjoyment and lightness in their everyday lives [1]. Collectively, current literature underscores the need to develop interventions that enhance balance in everyday activities and underpin enjoyment and quality of life despite life-limiting illness.

To date, neither rehabilitation nor palliative care efforts have focused on ensuring a balanced mix of everyday activities for this group of people or on how enjoyment can be facilitated [13, 14]. Only a RTC by Nottelman et al. reported a borderline effect on quality of life of a palliative rehabilitation intervention in people with advanced cancer and their relatives [15]. The intervention consisted of two mandatory consultations with a palliative physician and a nurse and a 12-week group-based intervention programme comprising a patient/caregiver school and individual physical training [15]. The patient/caregiver education included sessions, which lasted approximately 1 h where patient and caregiver exchanged personal experiences with different topics like fatigue, body and movement, coping with the patient role and rest and relaxation. The participants reported that the group-based intervention with physical exercise and symptom education made a positive difference in their social and physical well-being [15]. The intervention, however, focused on problems rather than resources and thus failed to pay attention to the elements that contribute to enjoyment in everyday life. A RCT study by Gomersall et al. evaluated an individually tailored, text message-enhanced rehabilitation intervention and found it to be feasible and acceptable in people with cancer and survivors [16]. However, the focus was predominantly on promoting physical activity behaviour than supporting a better balance in everyday activities and providing enjoyment in their everyday life [16]. Two other RCT studies aiming to enhance everyday activities demonstrated that prioritisation of resources and activities was among the most frequently used intervention components among persons with advanced cancer [17, 18]. In order to underpin an expedient balance of everyday activities and resources, it is decisive to change daily activity patterns (habits and routines) [19] so that activities that enhance enjoyment and quality of life may be prioritised. Additionally, extensive evidence shows that physical activity may enhance energy levels, increase physical capacity and enhance quality of life [20, 21]. To our knowledge, no previous studies have reported on interventions that support a better balance in everyday activities in people with advanced cancer.

An intervention for people with advanced cancer that targets balance in everyday activities calls for a complex intervention that integrates rehabilitation and palliative care principles by ensuring that the patient’s functional level is maintained while also providing the required relief and support [22, 23]. The Medical Research Council (MRC) guideline describes a circular and iterative process comprising four phases: (1) development, (2) feasibility, (3) evaluation and (4) implementation [19]. Involvement of stakeholders in all phases is recommended in order to maximise the intervention’s potential impact [24]. We developed version 1.0 of a resource-oriented palliative rehabilitation intervention. This intervention combines rehabilitation and palliative care principles and was developed based on extant research [15, 17, 18, 20, 21, 25], existing clinical experiences [1, 26] and input from a panel of users including people with cancer and advanced cancer and relevant healthcare professionals.

The present paper outlines the protocol for a feasibility study that will test contents and delivery of a resource-oriented palliative rehabilitation intervention in people with advanced cancer [27]. The following questions will be addressed:

### Contents and delivery of the intervention

1. Which intervention sessions are particularly relevant in the experience of the participants and the healthcare professionals?
2. How do the participants experience, interact and respond to receiving a resource-oriented palliative rehabilitation intervention?
3. Fidelity, adherence, dose and reach of version 1.0 of the intervention manual
  - Fidelity: Do the healthcare professionals deliver the intervention as planned? And do the healthcare professionals find that they have sufficient knowledge about the intervention?
  - Adherence: Are the participants able to participate in the intervention (the individual sessions)?
  - Dose: Which sessions were offered and what was the overall time expenditure?
  - Reach: What characterises the group that received the intervention?
4. Are changes observed in the participant’s quality of life, balance in everyday activities, physical function and fatigue after having received a resource-oriented palliative rehabilitation intervention?

### Outcomes and instruments

5) Which outcomes were not completed sufficiently (missing data)?

## Methods/design

### Trial design and setting

A feasibility study with a repeated-measurement design without a control group will be conducted at the research clinic of REHPA, the Knowledge Center for Rehabilitation and Palliation (in Danish language: REHPA, Videncenter for Rehabilitering og Palliation). REHPA is part of Odense University Hospital, Denmark. The REHPA research clinic offers residential intervention stays for people with life-threatening illness. A residential intervention stay typically consists of a 5-day stay followed by a 2-day follow-up stay approximately 12 weeks later. Since the feasibility study is to be conducted at the REHPA research clinic, a similar structure for the intervention informed the present study. We also know from existing research that 12 weeks of intervention are appropriate for people with advanced cancer since they have a shorter life expectancy [15, 17, 18]. The clinical staff at REHPA is comprised, among others, of occupational therapists and physiotherapists, nurses, physicians, social workers and psychologists. In May 2021 and October 2021, two innovative stays were offered that are different from the standard stays otherwise offered by REHPA [26]. In these innovative stays, the present resource-oriented palliative rehabilitation intervention was tested among people with advanced cancer. Standard Protocol Items: Recommendations for Interventional Trials (SPIRIT statements) were employed in the preparation of this protocol [28].

### Eligibility criteria

Participants need to meet the following inclusion criteria:

- Adult (≥ 18 years) and residing in his or her own home
- Has chronic or advanced cancer
- Experiences a need for support to manage everyday activities and to enhance the balance between various activities and tasks of daily living, e.g. enhance the balance between necessary activities and activities that produce enjoyment and meaningfulness
- Is able to participate in the course and willing to complete questionnaires and participate in interviews
- Must be independent with respect to personal activities of daily living (personal care, dressing and eating)
- Speaks and understands Danish language

### Recruitment

REHPA will prepare participant information, which will be sent to all palliative teams at hospitals, relevant municipalities, patient associations and cancer counselling services. In addition, the participant information will also be made available on REHPA’s web site and social media like LinkedIn and Facebook. The general practitioner or oncologist at the hospitals will assess potential participants based on the above-listed inclusion and exclusion criteria and will refer them to a REHPA stay if they fulfil these criteria. A responsible clinical healthcare worker will then in collaboration with the group of researchers decide who is eligible for a stay. Potential participants will receive detailed verbal and written information about the study. Prior to inclusion, the study participants are to provide written informed consent.

### Interventions

The “template for intervention description and replication” (TIDieR) checklist will be used to describe the intervention [29]. The intervention will be implemented as two residential REHPA stays consisting of a 5-day stay with subsequent half-way contact and a 2-day follow-up stay after 6 weeks.

The intervention will target peoples’ resources, enhance balance in everyday activities and underpin enjoyment and quality of life. The intervention is structured much like standard REHPA stays with a combination of group presentations and activities [26]. In order to underpin the focus on balance, activity, enjoyment and quality of life, the intervention comprises the following sessions:

1. Introduction to activity, balance and everyday life
2. Introduction to “Walk to get happy”— activities in nature
3. My everyday routine and activities: introduction to diaries
4. Balancing resources, fatigue and energy — how to?
5. My everyday life — balance, challenges and enjoyment
6. “Walk to get happy” — activities in nature
7. Life in movement
8. Yoga
9. Meaningful activities: what makes you happy?
10. Creative expression
11. Relaxing massage: “be good to yourself”
12. Values and action plan
13. “Developments since the previous session”
14. Life in movement — family, friends and network
15. Body and movement

**Table 1:** Description of a resource-oriented intervention

Intervention features	Intensity and contents
Setting	Residential stay at REHPA
Format	Primarily group based
Intervention provider	Multidisciplinary
Number session	15 mandatory and 1 optional
Intervention period	7 days
Time per session	45 min. to 2.5 h
Telephone follow-up	1

Thus, a total of 15 sessions are planned during the 5-day stay and 2-day follow-up stay combined. Each session has a duration of 45–150 min. Additionally, two individual conversations are offered at both stays and one optional session during the 2-day stay, the contents of which is determined by the participants. An optional half-way follow-up telephone contact is also offered to the participants. The sessions are imparted by a multidisciplinary team consisting of a nurse, an occupational therapist, a physiotherapist, a medical trained artist, a mindfulness coach and a social worker. The intervention is primarily group-based, and all 15 sessions are mandatory (See Table 1). If participants are absent from the mandatory sessions, this will be recorded as not being adherent to the protocol and will be registered in an intervention logbook (more information is provided under data collection). See Table 2 for more details about the sessions. At the 2-day follow-up stay, an optional session is offered to the participants of which they determine the content. On the last day at the 5-day stay, a healthcare professional asks the participants about their wishes to the content of the optional session.

**Table 2:** Details about the sessions

Mandatory sessions and form	Day		Time and provider
**Session 1** Introduction to activity, balance and everyday life: group based	1	This session provides an overall introduction to the intervention and the significance of everyday activities for people’s daily life, health and well-being. Furthermore, the session also provides knowledge about the importance of having appropriate balance of different types of everyday activities in daily life The contents consist of the following: • Introduction to the residential stay • Introduction to the concept of everyday activities, i.e. activity, health and well-being and balance in activities	45 min Occupational therapist
**Session 2** Introduction to “Walk to get happy” — activities in nature: group based	1	This session aims to provide the participants with knowledge about walking and physical activity as important factors to improve physical and mental health. In addition, it adds that nature as context can increase happiness/enjoyment and well-being The contents consist of the following: • Introduction to walk as an activity and the use of nature as a source for obtaining increase energy and happiness/enjoyment • Provide knowledge about physical activity in nature and how to prioritise this in daily life to achieve happiness/enjoyment and well-being • Provide knowledge about how to integrate physical activity in daily life	45 min Physiotherapist
**Session 3** My everyday routine and activities — introduction to diaries: group-based	2	The session introduces the participants to diary as a method to get insight into their everyday activity pattern The contents consist of the following: • Introduction to diary and its usefulness • In pairs, they discuss their filled-out diary during 1 day of activities • Plenary discussion about the diaries *Material: time-geography diary method during 1-day activities*	60 min Occupational therapist
**Session 4** Balancing resources, fatigue and energy — how to? Group based including lectures, discussions and individual assignments	2	The session provides the participants with strategies to better balance resources and energy in everyday life They get knowledge about the following: • Fatigue: use of breaks, activity adaptation and positioning • How to plan and prioritise activities bringing meaning and enjoyment: introduction to energy schema and score • Assistive devices: guidance in application hereof *Material: energy schema and score*	60 min Nurse
**Session 5** My everyday life — balance, challenges and enjoyment: group based	2	The session combines the achieved knowledge from sessions 3 and 4 and provides the participants with more in-deep knowledge about ways in which to improve their activity balance, i.e. change their activity pattern so that it includes a mix of activities regarding chores, social activities and relations, physical activities, creative activities and general activities bringing enjoyment The contents consist of the following: • Reflections upon the participants’ activity balance — are they satisfied? • Use activities to better achieve activity balance • Introduction to and work with activity wheel as method to achieve activity balance *Material: The activity wheel is a circle illustrating which activities the participants have performed and how much time they have usted on those activities. It is a visual around the clock illustration of their everyday activity balance*	60 min Occupational therapist and nurse
**Session 6** “Walk to get happy” — activities in nature: group based	2	In this session, the participants engage in doing different movements and physical activities in the form of games, indoor and outdoor in nature The contents consist of the following: • Doing physical activities in nature, walk and movement games • Doing physical activities indoor	75 min Physiotherapist
**Session 7** Life in movement: group and individual based	3	The session explores and starts a reflection among the participants on what contributes to a meaningful life and involves them in brainstorming on ideas to implement these meaningful aspects in their daily life The contents consist of the following: • Introduction to sources of meaning • Individual work where the participants choose 3–5 cards that symbolise important and meaningful things • Group discussion based on the cards • Ideas on how sources of meaning can be a larger part of their daily life *Material: Sources of meaning card method*	150 min Psychologist
**Session 8** Yoga: group based	3	The session introduces the participants to yoga and try out breathing and relaxation exercises. The participants will obtain more knowledge about how yoga can be used to experience stress relief both physically and mentally The contents consist of the following: • Warm-up focusing on movement and breathing • Doing meditation and breathing exercises • Doing yoga exercises • Stress relief	90 min Physiotherapist and certified yoga instructor
**Session 9** Meaningful activities; what makes you happy? Group based	4	The session provides knowledge of and starts a reflection among the participants about how meaningful activities have changed during their course of life, for instance because of illness. The participants get insights into their meaningful activities and what contributes enjoyment and happiness. They will also reflect upon which everyday activities should be part of their future daily life The contents consist of the following: • Short presentation about the meaning of everyday activities through phases of life • The participants engage in reflective teams where they discuss and present which everyday activities should be part of their future narrative using a timetable filled out before the session • They get peer-to-peer support based on their narrative with new everyday activities *Material: Timetable with the participants meaningful activities*	60 min Occupational therapist
**Session 10** Creative expression: group based	4	The session introduces the participants to creative activities as means of alleviating suffering and diverting attention from illness and problems The contents consist of the following: • Doing simple mindfulness exercises in preparation for the creative activity • Doing collage of important aspects of their daily life	150 min Medical trained artist and mindfulness coach
**Session 11** Relaxing massage “Be good to yourself”: individual based	4	This session offers relaxing massage to provide rest, well-being and more energy to the participants The contents consist of the following: • Relaxing massage using soft and dynamic grips	45 min Massage therapist
**Session 12** Values and action plan: group based	5	This session introduces a plan of action to implement the new strategies and everyday activities into their daily life when returning home. They will also return to what they value and how this can be a larger part of their daily life The contents consist of the following: • Discuss values • Set goals and define wishes for their daily life • Group discussions about the plan of action	60 min Social worker and occupational therapist
**Session 13** “Developments since the previous session”: group based	6	This session follows up on session 12 regarding how the participants have worked with and succeeded with their goals and changes after the 5-day residential stay The contents consist of the following: • Group discussions about achieved goals and changes in their daily life • How have these goals and changes affected their quality of life	60 min Social worker
**Session 14** Life in movement — family, friends and network: group and individual based	6	This session focuses on social relationships and particularly on belonging as an important part of experiencing meaning in life. The session therefore supports the participants in being beware of their social network of family, friends and other kind of persons in their lives The content consists of the following: • Introduction to social relationships and changes after life-threatening illness • The participants individually brainstorm on which persons should be in their social network and how to be closer to them in their daily life • Plenary discussion of advice and actions that may be taken to accomplish a more fruitful social network	90 min Psychologist
**Session 15** Body and movement: group based	7	In this session, the participants again engage in doing physical activities that bring enjoyment and happiness The contents consist of the following: • Doing physical activities indoor and outdoor	30–90 min Physiotherapist
**Optional session**	7	In this session, the contents are determined by the participants	90 min
**Evening activities**			
Music and singing	1		90 min Professional singer
Everyday life and existence	2		90 min Priest

Furthermore, the participants will also be offered to participate in the following evening activities:

- Music and singing
- Everyday life and existence

The evening activities are not sessions part of the present resource-oriented palliative rehabilitation intervention but are optional activities that are offered to the participants two times during the 5-day stay. The evening activities are led by a professional singer and a priest. These activities have a duration of 1 and half hour.

In the beginning of the course, the focus is on getting to know each other and learning about each other’s everyday life. Subsequently, the participants become engaged in various activities. Towards the end of the course, an action plan is prepared in dialogue between the professionals and the participants describing how they can implement the elements that produced enjoyment in their everyday lives when they return home. The preparation of the action plan will, among others, be based on the participants’ diaries and their own priorities of what they want to do and how they prefer to structure their everyday lives to ensure sufficient energy and time for the activities that provide enjoyment and improve their quality of life. For that purpose, the time-geography method is used [30]. This involves working with a structured diary where everyday activity patterns are recorded along with perceptions of enjoyment and values, productivity and rest and the balance between these elements [30]. The diary needs to be completed before the 5-day stay and is used in session 3.

### Theoretical framework for the intervention

The overall theoretical framework for the intervention is the WHO’s definition of palliative care [31] and the white book on the concept of rehabilitation (in Danish language) [32]. While palliative care focuses on relief of suffering, rehabilitation focuses on functional ability. Both concepts share the aim of enhancing quality of life [31, 32]. More specifically, the preparation of the intervention is inspired by the American philosopher, psychologist and learning theorist John Dewey [33]. Dewey describes learning as a social process of interaction that involves continuous transaction between action, activity and experience, where learning is furthered by the interplay between presentation/introduction of knowledge and experience-based perception [33]. Following Dewey, the acquisition of new knowledge/learning embraces the following five aspects: (1) experiences with activities that are relevant and of interest for the person, (2) challenges that are perceived as real/relevant, (3) introduction to necessary and relevant knowledge, (4) possible action strategies must be acknowledged or developed by the person, and (5) it must be possible to test ideas through action/activity and to bring them to practical use. On this basis, the intervention is established through interaction between sessions consisting of presentation of knowledge and workshops allowing the participants to test and engage in different activities. Additionally, each individual session is informed by theory about the topic in question [19, 26, 34, 35] and by knowledge from several of the previously mentioned studies that have combined/coordinated rehabilitation and palliative care [15, 18]. See Table 2 for more detailed description of the intervention.

### Context/location

The intervention is implemented through stays at the REHPA research clinic, located on the 3rd floor of Nyborg Hospital, Denmark. The clinic was designed in accordance with principles of ‘architecture and relief”, including individual rooms with a shower and rest room for the participants. Furthermore, a dining room and two living rooms are available. The ground floor includes a gym, a classroom, a reception (the welcome area), a café area and several group rooms. REHPA is located in a recreational area characterised by natural beauty and easy access to walking or running, etc. by the ramparts of Nyborg Castle [26].

### Data collection

Data will be collected at baseline (T1), during the 5-day residential stay (T2), after the 5-day stay has concluded (T3), before the follow-up stay at 6 weeks (T4), during the follow-up stay (T5) and after 12 weeks of follow-up (T6). Data at T2 and T5 is collected after each session for all participants. The following data collection methods will be used: (1) questionnaires, (2) focus group interviews and (3) participant observations.

### Outcomes

Since the intervention aims to enhance the participants’ quality of life and balance in everyday activities through prioritisation of energy and strength, engagement in physical and meaningful and joyful activities, the following outcomes will be chosen: (1) quality of life, (2) balance in everyday activity, (3) physical function and (4) fatigue, collected at T1, T3, T4 and T6. The outcome questionnaires will be sent and filled out electronically by the participants using the Civil Registration System in Denmark and the Research Electronic Data Capture (REDCaP). Two written reminders will be sent 3 and 6 days after deadline. Then, a reminder will be conducted by phone.

### Quality of life, physical function and fatigue

Quality of life, physical function and fatigue are measured using the European Organisation for Research and Treatment of Cancer Quality of Life Questionnaire Core-30 (EORTC QLQ-C-30). The EORTC QLQ-C-30 is a cancer-specific questionnaire containing 30 questions that address function as well as symptoms and quality of life. Answers are scored on an ordinal scale ranging from 1 to 4 (1 = not at all, 2 = a little, 3 = quite a bit, 4 = very much). Additionally, the questionnaire measures quality of life using an ordinal scale ranging from 1 to 7. Subsequently, the ordinal data are transformed into a score ranging from 0 to 100, where a higher score equals better function and higher quality of life or poorer experienced symptoms. The EORTC QLQ-C-30 was found to be valid, reliable and associated with high response rates among persons with advanced cancer [36].

### Balance in everyday activities

The Occupational Balance Questionnaire (OBQ) is a generic questionnaire comprising a total of 11 items that provide an overall assessment of balance in everyday activities. The items assess in different ways the satisfaction with the amount of and variation in various everyday activities in which people become engaged. Each question is scored on a 4-step ordinal scale ranging from 1 = completely disagree to 4 = completely agree. Based on the 11 questions, a sum score ranging from 11 to 44 is calculated. A higher score indicates a better balance in everyday activities. The QBQ has be found to be valid and reliable [37, 38].

### Quantitative feasibility data

An intervention logbook will be prepared that collects data about: (1) dose (duration and number of intervention sessions given) (T2 and T5), (2) if the contents were delivered as planned (fidelity) (T2 and T5), (3) if the participants were able to participate in the intervention sessions (adherence) (T2 and T5) and (4) the relevance of the intervention sessions (T2 and T5). The intervention logbook will be developed based on the guidance by O’Cathain et al. about feasibility studies [27]. We also record how many participated in the half-way follow-up. The healthcare professionals from the REHPA collect data from participants and among themselves and also state which intervention sessions were particularly relevant. All of these data are collected in the course of T1-T6 (see Table 3).

**Table 3:**
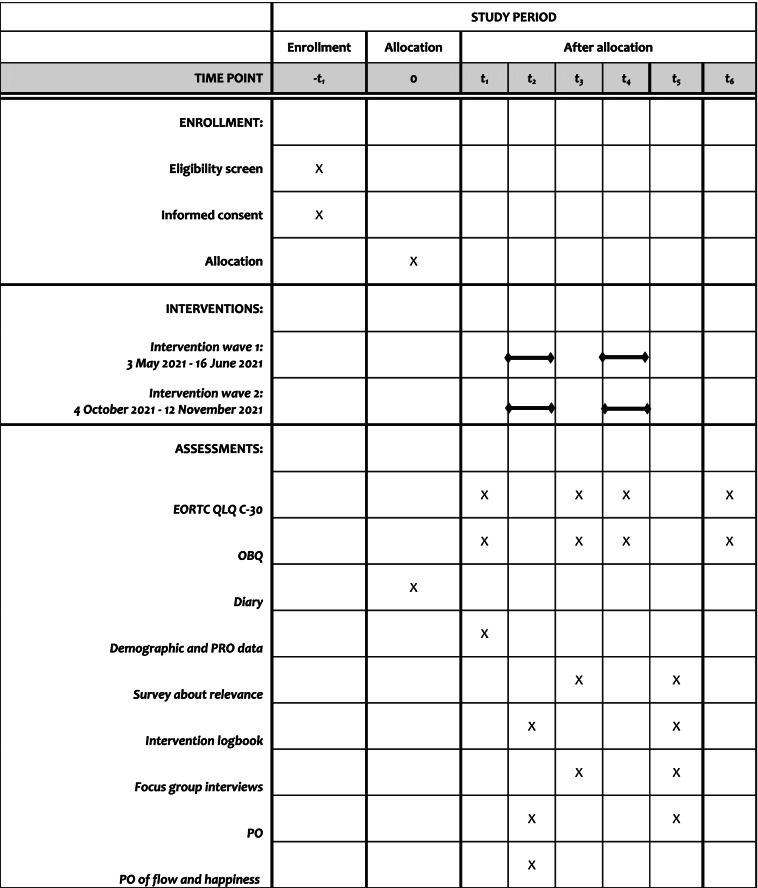
Time flow

*EORTC QLQ C-30* European Organisation for Research and Treatment of Cancer Quality-of-Life Questionnaire Core-30, *OBQ* Occupational Balance Questionnaire, *PRO* Patient-reported outcome, *PO* Participant observations

Data on reach will be collected based on the REHPA’s “How are you?” questionnaire [26] in which both demographics and patient-reported outcome (PRO) data will be employed to describe the group that received the intervention. See Table 3 for overview of the time line.

### Qualitative feasibility data

Data on the participants’ experiences, interaction and responses to the intervention will be collected through participant observation and focus group interviews. Participant observation is conducted during the two stays to gain insight into the participants’ immediate reactions to and experience with the intervention and their interaction with the REHPA healthcare professionals. Furthermore, a structured observation table will be used to register expressions of flow and enjoyment while the participants are engaged in session 10. Focus group interviews will be conducted on the 5th day of the 5-day stay and again at the second stay in smaller 5–7 participant groups [39].

A focus group interview will be conducted with the healthcare professionals who imparted the intervention in order to collect their experiences and perceptions from this type of intervention [39].

### Sample size

There are no requirements as to the number of participants needed in feasibility studies [40]. We deem that 20–30 persons will be sufficient to ensure that enough information about the contents of the intervention is collected and to explore potential changes over time in the chosen outcomes [40].

### Analysis

Continuous, normally distributed data are analysed by mean and standard deviation (SD). Otherwise, medians and percentiles will be used. Ordinal data are analysed by medians and percentiles, whereas categorical data and dichotomous data are analysed using numbers and percentages. The number of missing answers in the outcome instruments is calculated and presented descriptively as numbers and percentages. Wilcoxon signed-rank test will be used to analyse changes from T1-T3, T1-T4, and again from T1-T6 with respect to quality of life, balance in everyday activities, physical function and fatigue. Furthermore, a responder analysis will be conducted to determine how many of the participants have achieved a clinically relevant change (5–10 points) [36]. This analysis will focus on quality of life, physical function and fatigue, where it is possible to establish a clinically relevant difference [36]. The significance level will be ≤ 0.05, and 95% confidence intervals will be presented. Analyses will be performed using STATA 16.

Qualitative data will be transcribed verbatim from interviews and field notes and analysed using thematic analysis [41]. First, all texts from participant observations and focus groups will be analysed separately through an iterative process of reading, identifying themes, rereading and collapsing themes into distilled themes of the core findings. The preliminary findings from the first round of analysis of both participant observations and focus groups will be discussed to identify themes across the data material, and final themes will be identified. For the purpose of process analysis, the identified themes will be further qualified by relevant theory.

### Ethics

The study follows the principles of the Helsinki Declaration [42]. The scientific-ethical committee decided that no approval was required for this study (S-20210013). The study was approved by the Region of Southern Denmark Data Agency (R. no. 21/13073) and registered at ClinicalTrials.gov (NCT04772690). Data from the research database — rehabilitation and palliative care for cancer patients and others with life-threatening illness — have been approved and recorded with the Region of Southern Denmark: R. no. 18/27843. Oral and written consent will be obtained from all participants. Data will be collected electronically and stored in the REDCaP, a safe database administered by the Region of Southern Denmark. Any forms not collected electronically will be scanned and placed on a safe Sharepoint site. This also applies to audio files from the focus group interviews and written transcripts.

## Discussion

This protocol for a feasibility study presents a new approach in rehabilitation and palliative care for supporting people with advanced cancer. Instead of a predominant focus on functional problems and suffering, the present resource-oriented palliative rehabilitation intervention will target the participants’ resources, enhance balance in everyday activities and underpin experiences of enjoyment. In particular, leisure activities seem to be bring enjoyment for people with advanced cancer while at the same time they can support functioning [43]. It is therefore important to assist people with advanced cancer in prioritising their energy on leisure activities rather using all their resources on self-care activities [3, 4], although these kind of activities also serve an important purpose [8]. The focus on a balanced mix of everyday activities is pivotal and may improve quality of life [2, 44]. From a theoretical perspective, balance in everyday activities is important as all human beings need a variety of everyday activities that offer desirable levels of pleasure, productivity and restoration [35]. Still, a crucial point seems to be the balance itself between activity on the one hand and restituation and relief on the other. While attention towards the positive aspects of life through a focus on resources and enjoyment may be highly needed for people with advanced cancer [1, 12, 43], it is equally important not to ignore the potential risk of overlooking the difficult and painful challenges of these peoples’ circumstances, requiring relief and supportive care.

The MRC guide recommends drawing on both clinical expertise and evidence when developing a new intervention [22]. The evidence base of the resource-oriented palliative rehabilitation intervention draws mainly on knowledge from the Cancer Home-Life Intervention [12, 18, 45–47] together with evidence about creative [11, 48] and physical activities [20, 21]. The Cancer Home-Life Intervention is an occupational therapy-based intervention aiming at enabling people with advanced cancer to perform and participate in the everyday activities at home that they prioritise but have difficulties performing [47]. The newly updated MRC framework has added some core elements to the existing model of how to develop and evaluate complex interventions. These elements are as follows: context, programme theory, identify key uncertainties, refine intervention, economic considerations and involvement of stakeholders. These elements should be considered throughout all the phases [24]. In particular, it may be highly important to involve stakeholders early in the development phase [24]. However, involvement of stakeholders requires careful consideration of how to identify and engage them in the process [24]. We identified stakeholders among REHPA’s user panel of people with life-threatening illness including advanced cancer. We presented the initial ideas to these stakeholders and obtained their opinions about the suggested ideas. Co-production is a recommended approach to intervention development, which means that relevant stakeholders are involved in the decision-making process together with the researchers [49]. Professionals who were involved in the intervention delivery were also consulted about the ideas, and they had the same level of decision-making powers as the researchers [49]. In co-production with the researchers, the professionals developed the manual for the present resource-oriented palliative rehabilitation intervention. Co-production may have pros and cons; it may reduce the gap between research and clinical practice and thus later encounter fewer implementation barriers [49]. The downside may be that it can affect the evidence base if the professionals’ points of view are getting to much influence on the contents, as some experiences might be based on old and ineffective ways to intervene [50]. Nevertheless, the value of the practitioners experience and knowledge is equally important as that of scientific evidence which accords the principle of evidence-based practice (scientific evidence, patient preferences and clinical knowledge) [51]. Overall, this illustrates the complexity and lengthy process when drawing on the MRC framework to develop and evaluate a complex intervention. However, the approach provides a robust and meticulous process from intervention development to implementation, which, in turn, can prevent important flaws and save resources.

Since the intervention is newly developed, several uncertainties remain. These uncertainties need to be tested and explored in a feasibility study before proceeding to a pilot and RCT study [24]. The present feasibility study will therefore contribute with important knowledge to further develop the contents of the resource-oriented palliative rehabilitation intervention. Following the present feasibility study, the next step will be to adjust the intervention and then perform a pilot study in Danish municipalities. The pilot study will test the final version of the intervention in the context in which it will be implemented. The main focus of the pilot study will be on methodological issues like recruitment and drop-out rate, randomisation and test procedures regarding data collection. Future perspectives are to evaluate the intervention for effect, process and cost-effectiveness.

## Data Availability

Data will be stored in REDCaP and on a safe Sharepoint site.

## Abbreviations

RCT: Randomised controlled trial
MRC: Medical Research Council
REHPA: Danish Knowledge Centre for Rehabilitation and Palliative Care
SPIRIT: Standard Protocol Items: Recommendations for Interventional Trials
TIDieR: Template for intervention description and replication
T1: Baseline
T2: Collection of data during the 5-day residential stay
T3: Collection of data after end of the 5-day stay
T4: Collection of data before the follow-up stay at 7 weeks
T5: Collection of data during the follow-up stay
T6: Collection of data after 12 weeks of follow-up
PRO: Patient-reported outcome data
OBQ: Occupational Balance Questionnaire
SD: Standard deviation
REDCaP: Research Electronic Data Capture
EORTC: European Organisation for Research and Treatment of Cancer
